# Supplementary material for: Granulocyte-colony stimulating factor controls neural and behavioral plasticity in response to cocaine
Source: Nat Commun. 2018 Jan 16;9:9. doi: 10.1038/s41467-017-01881-x (PMC5770429; doi:10.1038/s41467-017-01881-x)
Supplement: Supplementary file 1 — Supplementary Information [file 41467_2017_1881_MOESM1_ESM.pdf]

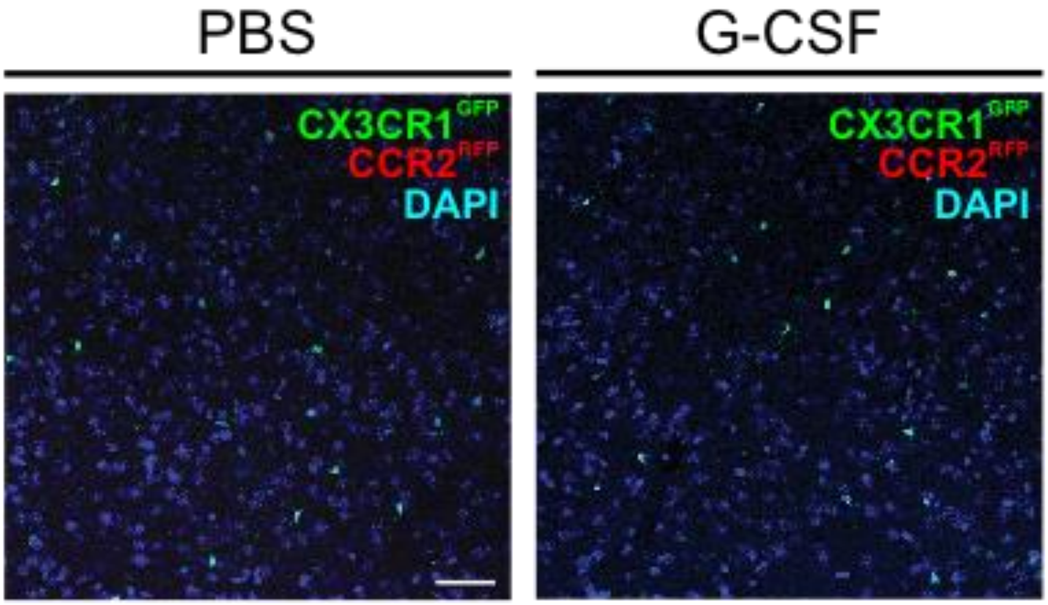

Supplementary Figure 1. Detection of CX3CR1- and CCR2-positive cells in the NAc of $CX3CR1^{GFP}$/$CCR2^{RFP}$ mice after treatment with G-CSF. No infiltration of CCR2-positive peripheral immune cells was observed following 7 daily injections with G-CSF or PBS. Representative confocal images acquired in the shell of the NAc. Nuclei were counterstained with DAPI, RFP-fused CX3CR1 protein is detected in red, and GFP-fused CCR2 is detected in green. Scale bar = 50 µm.

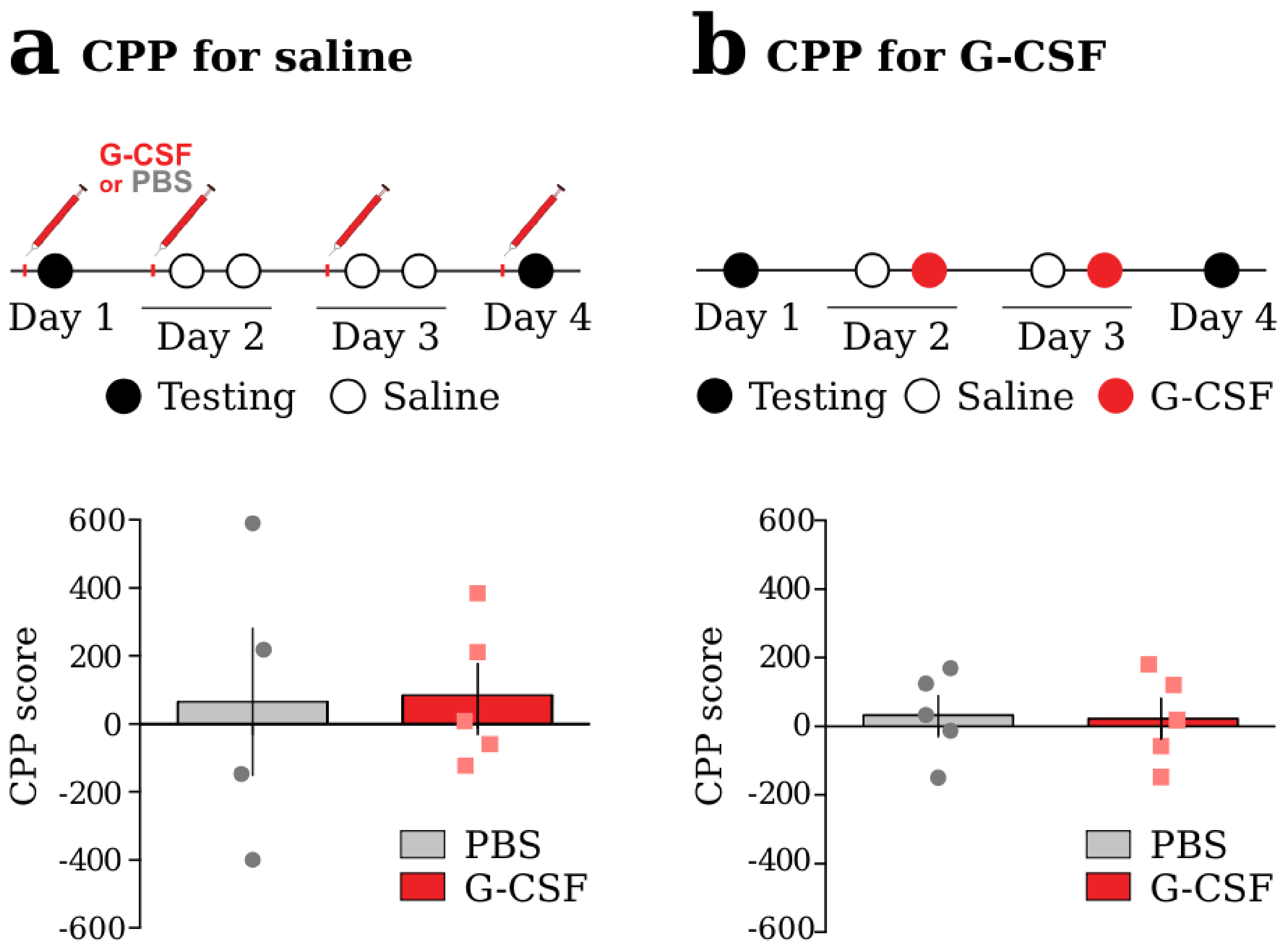

Supplementary Figure 2. G-CSF does not create any preference or aversion. a) To ensure that G-CSF does not elicit any preference or aversion on its own, animals (n = 5 per group) were injected with G-CSF (50 µg/kg) or PBS each morning prior to pairings of saline injection in both chambers. These animals did not form any preference for a chamber (Student's *t*-test; $t_{(7)} = 0.0862$, $p = 0.93$). (**d**) To test whether G-CSF could be rewarding by itself, we performed CPP (n = 5 per group) with pairing to a G-CSF injection (50 µg/kg) in one chamber. G-CSF pairings resulted in no preference or aversion ($t_{(8)} = 0.125$, $p = 0.90$).

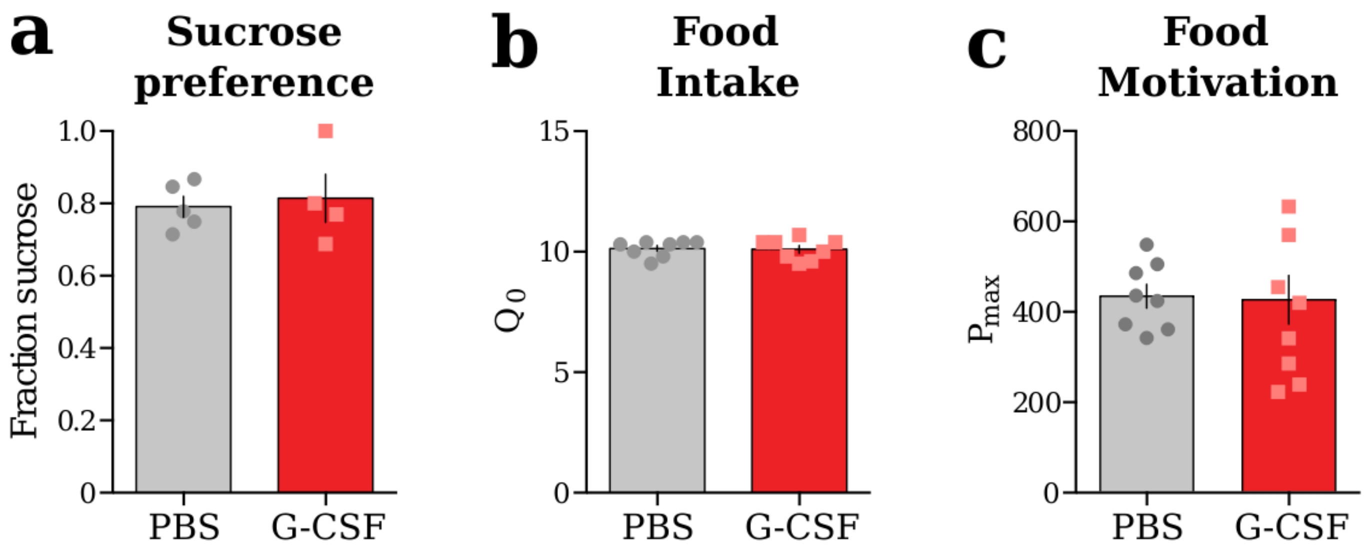

Supplementary Figure 3. G-CSF did not affect natural reward-related behaviors. (**a**) G-CSF (50 µg/kg, i.p.) had no effect on sucrose preference in a two-bottle sucrose preference task with mice (Student's *t*-test; $t_{(7)} = 0.348$, $p = 0.74$). (**b,c**) In a behavioral economics threshold task for food reward, rats injected with G-CSF (50 µg/kg, i.p.) had similar Q_0 (**b,** Student's *t*-test; $t_{(14)} = 0.1919$, $p = 0.85$) and P_{max} (Student's *t*-test; $t_{(14)} = 0.1285$, $p = 0.90$) values as compared to PBS-treated controls.

Gene target	Forward	Reverse
c-Fos	CTGGCAATAGCGTGTTCC	CAGACCACCTCGACAATGC
Csf3	TATAAAGGCCCCCTGGAGCTG	GCTGCAGGGCCATTAGCTTC
Csf3r	GTTTTGTGGGGAGTGGGGAT	TAACGCGGTGCTTGTCACTA
Gapdh	TTGTCAGCAATGCATCCTGCACCACC	CTGAGTGGCAGTGATGGCATGGAC
mCherry	GATAACATGGCCATCATCAAGGA	CGTGGCCGTTCACGGAG

Supplementary Table 1 – Primer pairs used for qPCR
