# Supplementary material for: Granulocyte-colony stimulating factor controls neural and behavioral plasticity in response to cocaine
Source: Nat Commun. 2018 Jan 16;9:9. doi: 10.1038/s41467-017-01881-x (PMC5770429; doi:10.1038/s41467-017-01881-x)
Supplement: Supplementary file 3 — Description of Additional Supplementary Files [file 41467_2017_1881_MOESM3_ESM.pdf]

File Name: **Supplementary Data 1**
Description: **Raw values of all analytes from cytokine multiplex.** Raw pg ml^{-1} values for each analyte in the cytokine multiplex ± s.e.m. The left side of the table presents the values from experimenter-administered saline and cocaine groups, and the right side of the table the self-administered saline and cocaine groups. The mean difference is the difference in means between the two groups. For each analyte, uncorrected *p* values from a two-tailed Student's t-test are also presented, with bold values being $p < 0.05$.

File Name: **Supplementary Data 2**
Description: **Correlation matrix of serum cytokine levels and behavioral response to cocaine.** Full listing of the Pearson's r correlation value for each cytokine with the extent of locomotor sensitization (left) and amount of cocaine intake (right). Uncorrected *p* values for each correlation analysis are paired with the r values, and items in bold represent $p < 0.05$.
